# Advancing Integrated Care through Practice Coaching

**DOI:** 10.5334/ijic.4737

**Published:** 2020-06-26

**Authors:** Benjamin F. Henwood, Elizabeth Siantz, Kimberly Center, Gale Bataille, Elise Pomerance, Jennifer Clancy, Todd P. Gilmer

**Affiliations:** 1 Suzanne Dworak-Peck School of Social Work, University of Southern California, Los Angeles, CA, US; 2 Department of Family Medicine and Public Health, University of California San Diego, La Jolla, CA, US; 3 Inland Empire Health Plan, Rancho Cucamonga, CA, US

**Keywords:** practice facilitation, qualitative methods, complex care

## Abstract

**Introduction:**

Practice coaches are skilled consultants who work in health care service delivery systems to make changes designed to improve patient outcomes, yet research is limited regarding their use to support integrated health care. This article describes the use of practice coaches in a large-scale effort to implement integrated care in the United States for patients with complex care needs.

**Theory and methods:**

This immersive, qualitative project involved five implementation team members; eight practice coaches; and 77 staff members from 12 health care organizations. Semistructured interviews were recorded and transcribed verbatim. Thematic and content analyses were applied in multiple stages to understand the use of practice coaches.

**Results:**

Qualitative themes about the use of practice coaching in this initiative were: (a) development of “a very rich coaching model”; (b) moving from an organic to standardized coaching approach; and (c) coaches representing the “face of the initiative.”

**Discussion:**

A rich coaching model that includes an interdisciplinary coaching team can support integrated care transformation but challenges including finding highly qualified coaches and sustaining and disseminating the coaching model exist. Standardization was seen as a way to address such challenges.

**Conclusion:**

Practice coaches can provide individualized, hands-on guidance to support successful implementation of integrated care.

## Introduction

Practice facilitators, also known as practice coaches, are skilled consultants who work in clinical practice environments and health care delivery systems to support changes designed to improve patient outcomes. These individuals assist service providers and quality-improvement teams to develop the skills needed to adapt clinical evidence or new best practices to the specific circumstances of a given health care delivery environment [1]. Previous studies of practice facilitation in general medical settings have found that it can increase preventive service delivery rates, improve chronic disease management, support implementation of system-level improvements [2], and support medical practices in adopting evidence-based guidelines [3].

Although practice coaches have been previously used in general medical settings, research is limited about their use to support integrated physical and behavioral health care for patients with complex health conditions [4,5]. Existing studies have suggested that practice coaches in integrated care settings can improve implementation and organizational outcomes, such as increasing the degree of change, ability to make changes (also known as adaptive reserve), referrals to behavioral health, and number of patients seen by behavioral health providers. Yet these studies provided only a brief description of their coaching model and little in-depth information on the coaching or facilitation process [6,7,8].

This study sought to fill this gap in the literature by leveraging the strengths of qualitative methods to examine the use of practice coaches in a large-scale effort to implement integrated physical and behavioral health care for patients with complex care needs in the United States. The Behavioral Health Integration Complex Care Initiative (BHICCI) was launched in September 2015 by the Inland Empire Health Plan, a nonprofit, Medicaid-managed care health insurance plan in California. Over a 3-year period, the BHICCI supported practice transformation in 12 health care organizations across 30 clinical sites, with the goal of improving both physical and behavioral health outcomes for patients with complex chronic conditions [9]. Participating clinical sites represent a diverse array of providers (e.g., federally qualified health centers, specialty behavioral health clinics, pain management clinics, a residential care facility) that have historically provided either physical healthcare or behavioral healthcare, but typically not both. For organizations that had both primary care and behavioral health clinics, these practices were not integrated and relied on referrals to the other when needed. Previously published results from this initiative found that the BHICCI improved rates of screening and clinical indicators for common chronic conditions including depressive symptoms, systolic blood pressure, hemoglobin A1C, and body mass index; increased patient satisfaction; and reduced costs in some settings [9]. An implementation evaluation also showed that participating clinical sites became substantially more integrated over the course of the project with the development of integrated care teams being mostly hindered by existing shortages in health care professionals.

Health plan executives and their key advisors developed the BHICCI approach to systems transformation based on the Institute for Healthcare Improvement’s (IHI) Breakthrough Series Learning Collaborative Model, which emphasizes the use of collaborative learning to achieve major changes in health care delivery [10]. Since it was determined that attempting to integrate physical and behavioral healthcare would be challenging and likely require individualized support in addition to semiannual learning sessions as part of the IHI model, practice coaching with was added to the model. Specifically, the health plan invested in eight practice coaches who had previous experiences working in clinics that had undergone a transformation process and were also experienced behavioral health clinicians or physicians (MDs). Practice coaches met monthly as a group with the health plan’s implementation team for planning purposes and to discuss the overall progress of the initiative. In general, each practice coach supported several care teams or health care organizations. Assignment of practice coaches was intentional and tailored to support unique transformation challenges. For example, to support practice changes in two of the large health care systems participating in BHICCI, practice coaches used a dyadic approach, which connected a coach who was a behavioral health clinician with an MD coach. Practice coaches provided individualized, hands-on guidance to support successful implementation of integrated, chronic disease management, and person-centered health care. Practice coaches met with health care teams in person monthly for up to a half-day to foster collaborative relationships, provide guidance in key areas of practice transformation, and observe transformation progress. Practice coaches were also available to their teams through web-based meetings, email, and phone that varied in frequency (a minimum of once a month to several times a week) based on the phase of the project and individual participant preferences.

In this study, we examine how practice coaching was utilized in the BHICCI and whether this coaching approach was viewed as valuable to the project. We also consider whether this coaching model could be disseminated to other integrated care initiatives.

## Theory and methods

### Data collection

This study was part of a larger, independent evaluation of the BHICCI that examined clinical outcomes and integrated care implementation [9]. For the current study, the first two authors, who were part of a University-based research team hired to evaluate the BHICCI and who have extensive experience with qualitative research and an interest in integrated care and health services research, relied on an immersive qualitative approach. This included conducting one-on-one interviews with key stakeholders including implementation team members at the health plan and practice coaches, site visits to integrated care clinics, and follow-up phone interviews with clinical staff who participated in the site visits. This resulted in four main sources of data for this study that are described below.

The first was key stakeholder interviews with five implementation team members who included health plan executives and key advisers who were paid consultants. These semistructured interviews were typically conducted over the phone, lasted approximately 30 minutes each, and included questions such as: Can you tell me about the background and implementation of this project? What are the goals of the initiative? The second data source was semistructured interviews with eight practice coaches that lasted between 30 and 40 minutes each. These interviews were typically conducted over the phone and also addressed perspectives on the overall project design and intended goals, but they focused mainly on understanding practice coaches’ perspectives of their roles and responsibilities and their experiences with participating organizations. Questions included: Can you tell me about the role of the practice coach? What is working well in your organizations? The third data source was implementation site visits at 12 participating clinics that lasted between 2 and 6 hours and involved touring the clinic, observing team meetings, and conducting interviews with clinic staff. Clinic sites were purposively chosen using maximum variation sampling to capture clinics of different size, focus (e.g. behavioral health or physical healthcare settings) and population served (e.g. predominantly English versus Spanish speaking). In total, seventy-seven staff members from a variety of professional backgrounds and roles in their clinic, including executives, registered nurses, primary care providers, data mangers, substance abuse specialists, and mental health practitioners, were interviewed in-person during site visits, either in a one-on-one or group setting. A semistructured interview guide included questions such as: What are some of the challenges you have or are facing related to the BHICCI? What is working particularly well? If the BHICCI was starting over today, what would you change? The final data source was follow-up phone interviews with leaders from seven of the twelve clinics that participated in the site visits, These seven clinics were chosen because their initial sit visit occurred earlier on in the BHICCI (during the first half of the 3-year project period) so that the study team could meaningfully ask about change over time since the initial visit. These phone interviews lasted between 30 and 45 minutes between March and July 2018 during the final 6-months of the evaluation. Questions included: How have things changed since our initial site visit? Are there any lessons learned? How critical were practice coaches to the project?

Participants were purposively sampled to gain a comprehensive understanding of the implementation of this integrated care initiative. An information sheet rather than a consent form was provided to all study participants questions since questions focused on the delivery of services and we did not request any personal information from participants, which was the basis for the study being classified as exempt by the institutional review board at the first author’s university. Nobody declined to participate in the study. The first two authors, who were part of the initiative’s evaluation team, had ongoing contact with the project’s implementation team and practice coaches through regular quarterly meetings that they attended to help document the overall implementation of the project. All interviews were digitally recorded, transcribed verbatim, and entered into an online qualitative software system (i.e., Dedoose.com) to assist with analysis.

### Data analysis

Thematic and content analysis involving constant comparative methods was used to analyze the qualitative data for this study [11]. Analysis occurred in multiple stages that began with developing case summaries of key stakeholder and practice coach interviews that were reviewed by the first two authors to understand the overall design of the project and the coaching model and processes [12]. The second stage involved the development of a code book that was used to apply codes (e.g., implementation challenges, team meetings, working with practice coaches, and coordinating complex care) to transcripts from site visits [13]. Relevant coded material (e.g., working with practice coaches, implementation challenges) was then extracted and reviewed by the first two authors to better understand the role of practice coaches from program staff perspectives. The first two stages of analysis resulted in the development of initial set of themes that reflected the use of practice coaches in the overall initiative. These themes were then refined by the first two authors through a final stage of analysis in which a case summary matrix was developed to organize responses from site visit follow-up interviews and used for triangulation and completeness in presenting the final themes that were agreed upon by all authors [14]. Strategies of rigor related to qualitative methods employed during this study included prolonged engagement, team debriefing during data collection and analysis phases, independent thematic development, member checking, and consensus-driven findings [15].

## Results

Three emergent themes expanded our understanding of the use of practice coaching in this integrated care initiative: (a) development of “a very rich coaching model”; (b) moving from an organic to standardized coaching approach; and (c) coaches representing the “face of the initiative.” Illustrative quotes identified by study participant number (i.e., SP #) are provided throughout the text, with additional supporting quotes in Table 1.

**Table 1:** Illustrative quotes supporting thematic findings on practice coaching.

Theme 1. Development of “a very rich coaching model”

“I do think definitely this caliber of practice coach is critical, because the health care system is so full of really entrenched physicians and behavioral health clinicians, and so helping them to change definitely requires that they interact with people they respect, and docs only really respect docs, and they certainly don’t respect docs that are kind of coming at them that have just never been there and done that.” *–Expert consultant (SP 4)*
**Theme 2: Moving from an organic to standardized coaching approach**

“I think if there’s anything I would have thought of doing differently in a future project when it comes to practice coaches is just a little bit more of a standardization of the practice coaches.” *–Practice coach (SP 7)*
“The practice—the coach is probably the best word to describe it because your job is not to do their job. Your job is really to kind of look at what’s going on overall in terms of implementing the program, and you want to interface with the teams in a way that they feel encouraged and they feel like we have supports, but we’re not actually doing the work for them, because I think that the downside is if we did that then as soon as we left, they wouldn’t be able to do anything. So I think that’s always—that’s been a big balance to try to always think through before I say or do anything. Is this, what I’m going to say, going to be helpful for the team members to do their job?” *–Practice coach (SP 9)*
**Theme 3: Coaches representing the “face of the initiative”**

“Having the continuous practice coaching, especially early on, was crucial, because you really could get lost in the definitions of what this program is and how it should work and what the structure should kind of look like. And so we had flexibility, but I feel like without the practice coaches, things may not have been as successful, so I think the practice coaching was huge.” *–Health care provider (SP 33)*
“[Practice Coach] for me has been a great asset where she has plugged me in to all of the meetings that I need to go, all of the information that I need to read up on, what BHICCI is. She’s been very helpful. Anytime I have questions, I’ll email her and she gets back to me within 24 hours. So for me that’s been great, for her to plug me into all of these things that I need to know. So in that I feel very supported by her, where I know if I have any questions or require pretty much anything that I need, I know she will be there to help guide me.” *–Health care provider (newly hired primary care supervisor) (SP 54)*

### Development of “a very rich coaching model”

The approach to practice coaching in the BHICCI was regarded as innovative in two ways. First, the model was described by a health care executive as “a very rich coaching model” (SP 1) that employs highly credentialed senior experts in their respective fields. According to an expert consultant to the project, having highly qualified coaches was viewed as a defining feature of this model:

The practice coaches that [the health plan] approved were of a much higher level of education and experience than many of the practice coaches that are funded, for instance, through the CMS [Centers for Medicaid and Medicare] Innovations grants. (SP 2)

A second defining feature of the practice coaching model was that coaching teams featured experts in both behavioral health and physical health. To reflect the multidisciplinary nature of the intervention and communicate effectively with providers from medical and behavioral health disciplines, coaches were sometime paired such that one practice coach with a behavioral health background (usually a PhD or licensed clinical social worker) and one practice coach with an MD were assigned to a participating clinic. As one coach explained, “That’s another huge distinction, I think, in our coaching model from any other coaching model that I’m aware of, where you’ve got coaches that are partnered” (SP 6). Another coach acknowledged this approach: “I think it’s been really nice to have that mirroring of the multidisciplinary team approach in the practice coaching, I think it is really valuable” (SP 7). Having MD practice coaches in particular was viewed as an important part of the project because, as one MD practice coach explained,

I guess one way to put it would be to make the physician feel comfortable about the program by having someone who speaks the same language and that they intuitively would, I don’t know if trust is the right word, but as an understanding between physicians. (SP 10)

Although there was overall support from the health plan for this rich coaching model throughout the project, some of the coaches and project advisors questioned whether this model was necessary, sustainable, and scalable. As one of the coaches pointed out,

It’s like there is no good coaching manual out there that I found, quite frankly. You know, our model is more intensive than a lot of the coaching that’s out there. And the level of experience and skill of the coaches is actually higher … than many of the coaches’ models that I’ve seen. (SP 8)

A key advisor also commented that “this is such a new area that you don’t have any—very many people around the country that know how to do this” (SP 3). Most stakeholders agreed that to make the model more efficient and sustainable, increased standardization of coaching practices is needed.

### Moving from an organic to standardized coaching approach

When asked about their approach, coaches explained their work in various ways, but they all seemed to agree that it’s “more organic than cookie cutter” (SP 9). One coach described the process of coaching as “a combination of teaching and cheerleading and providing feedback. Balanced feedback” (SP 8). Individual coaches agreed that “there’s a consistency in not giving all the answers. But really helping people to find their own answers” (SP 11). Still, several coaches acknowledged that their role involved “coaching and content” (SP 12). Coaches said they felt they had a particular effect on team-based care, such as when an MD practice coach explained,

One thing that the PCP [primary care provider] never had experience with is how to sit and do a systematic case review, and so I would model that for them so explain how we do that … make it very focused, very outcome driven, data driven. (SP 13)

Several programs reported that practice coaches were especially supportive in helping teams consider team and patient experience in ways they had not previously. As one program reported:

[Practice Coach] gave us some information on the client experience, how they view our company is greatly affected by the front [desk] staff, because they spend the most time with the front staff, answering calls, making appointments, sitting in the waiting room. So we really wanted them to feel they’re a part of the team. They’re an important integral part of the team. (SP 32)

Still, coaches explained that it could be challenging to know when to offer that expertise. As one coach explained:

I’m just picturing when we’re out at the site trying to figure out what they’re ready to hear, what they’re ready to take on. There are so many details involved with this work. There are just so many areas to improve on, and so you can’t do everything at once, and so trying to identify what they’re ready to do today. (SP 7)

When working as a coaching team, coaches also acknowledged the importance of collaboration and communication with one another about their work with a particular clinic. As one coach explained,

What we’ll typically do is meet a little bit beforehand and at lunch we’ll touch base and then afterwards we’ll touch base to kind of debrief [about] what goes on when we meet the teams to make sure that we are thinking the same thing after a site visit. (SP 11)

Overall, it is also important to note the evolution in the coaching model during the project as the health plan’s expectations for practice transformation became clearer. As one key stakeholder explained,

Initially the coaches were all single agents and now they’re each paired with a medical doctor coach, so we moved to a buddy system and we’ve dramatically increased the number of weekly meetings. I mean, if you’re talking about as compared to when we first started, there’s now a two-hour weekly meeting for the coaches to promote standardization. (SP 4)

Standardization was viewed as a significant evolution of the coaching model and practices. As one coach reflected toward the end of the project, such standardization may imply that “you don’t need to be a licensed psychiatrist” (SP 12) and that a less rich model could be used in future projects.

### Coaches representing the “face of the initiative”

Although the practice coaches were outside consultants and not staff members from the health plan that supported the project, most stakeholders including health plan leaders, and participating clinics recognized that coaches “represented the face of the initiative” (SP 4) to the participating programs. When asked about the experience of working with practice coaches, one provider emphatically replied: “We need to keep her!” (SP 22). When asked about the importance of practice coaches, another provider expounded,

Critical. We are very blessed to have our practice coaches with us. We’ve had two that have really helped us along the way. Every time there was a question or something would come up, it was nice to have them available to be able to provide that support for us, so we wouldn’t feel alone, and we had that backbone, that support. (SP 27)

At least one provider regarded the role of the practice coaching as potentially more important than establishing a learning collaborative, which was viewed by most to be a major component of the practice transformation process. When asked about the critical nature of coaching and learning sessions, this provider explained,

It’s really been building the bridges with other clinics. So, getting to know other clinics and other organizations has been the biggest advantage, I think, for us. … But if you ask how critical they were for the whole process, I definitely would say that having a dedicated practice coach that checks in, in person, on a regular basis was way more important, or was more important than having this collaborative. (SP 53)

In addition to being supportive to individuals and providing information on program activities, many individuals also reported that they appreciated having an organizational liaison who could facilitate communication between their program and the health plan. In fact, liaising between the health plan and health care organizations was a major reason coaches became the face of the initiative. According to one behavioral health clinician: “They’re definitely our liaison. They’re the spokes that go out to the partners. If there’s an issue, they are the ones that would help craft how we might communicate that to [the health plan]” (SP 37). As liaisons, practice coaches also could report back to the integrated teams with “inside information” from the health plan. As one clinic provider explained, “She tells me what kinds of things are talked about in their meetings, in the coaches’ meetings, and what the focus is and what we need to do, and she provides feedback” (SP 61).

Overall, although there was near universal recognition of the value of having practice coaches, at various moments throughout the initiative, concerns about the coaching role were articulated. In a few cases, some clinic staff members admitted—as expected by the coaches—that they were initially skeptical of having support from a practice coach due to concerns that coaches would be overly controlling of their programs. Others expressed concerned that coaches were only there to monitor the teams.

Initially, I didn’t really, couldn’t really place them, and it was always like kind of like, “Are they here to control us? Are they here to make sure that we do what we’re supposed to do? Or are they really here to help us?” (SP 67)

One site reported moments when coaches gave inconsistent feedback that resulted in confusion on the part of the clinic staff:

You’d say, “You’re doing great, and just a suggestion, but do whatever you like, but it really has to be this way. But whatever you guys think would be good. … So, pick a model that works for you.” But if then they pick a model that works for them, then the next time it’s still critiqued, that just led to more confusion and frustration for them. (SP 75)

Despite some expression of frustration, such feeling never rose to the level of needing any conflict resolution between coaches and program staff. Other programs reported that practice coaches would have been more helpful if rather than serving as a liaison, they could provide more logistical support with issues such as how to set up a primary care practice (e.g., spatial layout, room size, regulations for storing medications that need refrigeration, etc.) for behavioral health programs attempting to integrate primary care.

## Discussion

The findings from this study suggest that for integrated care transformation efforts in the United States, a rich coaching model that includes an interdisciplinary coaching team with highly qualified and skilled practitioners is feasible and appreciated by front-line clinic staff members and health plan administrators alike. Bringing together expertise in both physical and behavioral health care systems was especially important because practice coaches needed to effectively communicate with providers from different backgrounds and have working knowledge of the political and administrative aspects of care systems that have historically been separated. This is consistent with the United States Agency for Healthcare Research and Quality’s recommendation that coaches have a master’s degree and professional health care experience [16], yet there are currently no set standards for practice coaching.

Our findings also suggest that if coaches do not have experiences with integrated systems, having a team-based coaching model may better support integrated care transformation. In addition, the relational elements of practice coaching may be especially important in the context of an integrated system because coaches support collaboration across different health care specialties and also help negotiate disciplinary differences, which is an aspect of coaching that is less emphasized in the literature [1,2]. In this case, the relational aspect of coaching was also important because coaches represented the face of the integrated care initiative and played a key role as the liaison between the sponsoring health plan and the individuals working in participating health care organizations. Being in such a role requires diplomacy and an ability to balance sensitivity to the needs of the health plan while serving as an ally to the individuals who staff community clinics.

Despite widespread support of this rich practice coaching model, several challenges consistent with the literature [17] were identified, including finding highly qualified coaches, the sustainability of such a coaching model, and the potential for the model’s wider dissemination. Increased standardization was viewed as a way of addressing such challenges, which could include outlining: (a) clear phases in the coaching relationship; (b) guidelines for dose, intensity, or titration of coaching over time; (c) logistics of team-based coaching; and (d) differences between core skills required of all coaches versus specialty coaching skills. These areas have all been included in a practice coaching manual that is currently being developed by the health plan that sponsored this initiative and should be considered by other systems that are planning to integrate care. Since the BHICCI initiative ended, practice coaching has continued but coaches are now employed by the health plan rather than being an outside consultant, which may help with issues of sustainability but could influence the relationship between coaches and clinic staff.

Although the strengths of this study include an immersive qualitative approach that captured multiple stakeholder perspectives, the findings should be viewed in light of some limitations. Most notably, we did not evaluate the effectiveness of coaching, nor did we assess whether the fit between coach and program affected the perspectives of either. We were also unable to determine when coaching was viewed as most valuable as we did not ask clinic staff to rank their experiences with coaches. It is also unclear the extent to which this practice coaching model would need to be tailored to healthcare systems outside of the United States.

## Conclusion

Practice coaches can provide individualized, hands-on guidance to support successful implementation of integrated care. Future research should continue to investigate the relational aspect of practice facilitation to improve the training of the coaching workforce and could assess the extent to which the fit between coach and program matters. Formal testing of a more a more standardized coaching model is needed and could be facilitated by the development of more formal coaching manuals that now exists from the BHICCI.
